# Patterns of helminth infection in Kenyan elephant populations

**DOI:** 10.1186/s13071-020-04017-1

**Published:** 2020-03-18

**Authors:** Edward King’ori, Vincent Obanda, Patrick I. Chiyo, Ramon C. Soriguer, Patrocinio Morrondo, Samer Angelone

**Affiliations:** 1 Department of Animal Pathology (INVESAGA Group), Veterinary Faculty, University of Santiago de Compostela, Lugo, Spain; 2 Veterinary Department, Kenya Wildlife Service, Nairobi, Kenya; 3 Institute of Primate Research, National Museums of Kenya, Nairobi, Kenya; 4 Estación Biológica de Doñana, Consejo Superior de Investigaciones Científicas (CSIC), Sevilla, Spain; 5 Institute of Evolutionary Biology and Environmental Studies, University of Zurich, Zurich, Switzerland

**Keywords:** Disease ecology, Epidemiology, Gastrointestinal parasites, Helminths, Nematodes, Trematodes, Wildlife

## Abstract

**Background:**

The dynamics of helminth infection in African elephant populations are poorly known. We examined the effects of age, sex, social structure and the normalized difference vegetation index (NDVI) as primary drivers of infection patterns within and between elephant populations.

**Methods:**

Coprological methods were used to identify helminths and determine infection patterns in distinct elephant populations in Maasai Mara National Reserve, Tsavo East National Park, Amboseli National Park and Laikipia-Samburu Ecosystem. Gaussian finite mixture cluster analyses of egg dimensions were used to classify helminth eggs according to genera. Generalized linear models (GLM) and Chi-square analyses were used to test for variation in helminth infection patterns and to identify drivers in elephant populations.

**Results:**

Helminth prevalence varied significantly between the studied populations. Nematode prevalence (96.3%) was over twice as high as that of trematodes (39.1%) in elephants. Trematode prevalence but not nematode prevalence varied between populations. Although we found no associations between helminth infection and elephant social groups (male *vs* family groups), the median helminth egg output (eggs per gram, epg) did vary between social groups: family groups had significantly higher median epg than solitary males or males in bachelor groups. Young males in mixed sex family groups had lower epg than females when controlling for population and age; these differences, however, were not statistically significant. The average NDVI over a three-month period varied between study locations. Cluster analyses based on egg measurements revealed the presence of *Protofasciola* sp., *Brumptia* sp., *Murshidia* sp., *Quilonia* sp. and *Mammomonogamus* sp. GLM analyses showed that the mean epg was positively influenced by a three-month cumulative mean NDVI and by social group; female social groups had higher epg than male groups. GLM analyses also revealed that epg varied between elephant populations: Samburu-Laikipia elephants had a higher and Tsavo elephants a lower epg than Amboseli elephants.

**Conclusions:**

Elephants had infection patterns characterized by within- and between-population variation in prevalence and worm burden. Sociality and NDVI were the major drivers of epg but not of helminth prevalence. Gastrointestinal parasites can have a negative impact on the health of wild elephants, especially during resource scarcity. Thus, our results will be important when deciding intervention strategies.
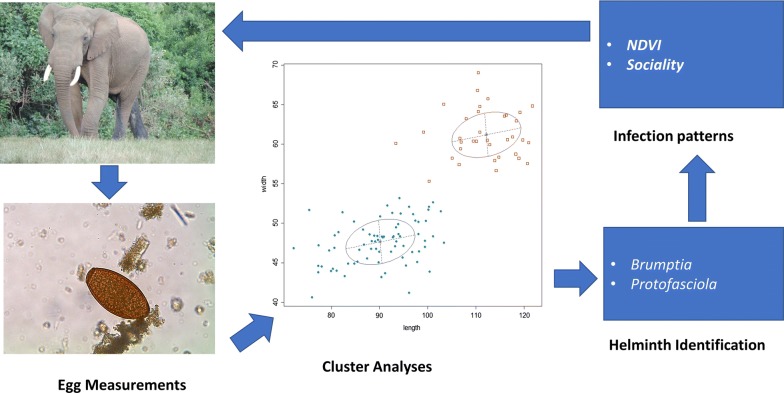

## Background

Most studies on the helminths parasitizing African elephants have in the past focused on helminth taxonomy and more recently on within population infection dynamics [1–4], but no studies have simultaneously examined inter-population and intra-population infection dynamics and their drivers. The most common helminths infecting African elephants are nematodes and trematodes; two groups of helminths that have environmentally dependent transmission mechanisms. Species of *Murshidia*, *Quilonia* and *Khalilia* are the most common nematodes infecting African elephants [5]. The free-living environmental stages of gastrointestinal nematodes are strongly affected by climate, e.g. extreme temperatures are detrimental to their development and survival. Moisture is needed for the development and transition of larvae from soil to pasture and so rainfall and vegetation may be limiting factors on transmission and may influence patterns of inter-population variability in infection patterns [6]. Some studies in Africa have found a significant positive correlation between mean annual precipitation (rainfall and relative humidity) and nematode infection rates [6–8]. Furthermore, some studies have also found associations between precipitation and certain qualitative measurements of egg burden (mean nematode species richness, mean number of nematode worms and infection intensity per individual host) [9–11].

Several species of trematodes are known to infect elephants [12] and some are associated with pathological lesions in starving animals [12, 13]. Trematodes of the family Fasciolidae usually have a complex life-cycle that involves a vertebrate host, in which they reproduce sexually, and an intermediate snail host, in which asexual reproduction occurs. The transmission of trematodes is largely driven by the presence of water and snails, suggesting that water availability and precipitation are important factors in their life histories [14]. Given the association between climatic factors and the propagation and transmission of both nematodes and trematodes, we used a normalized difference vegetation index (NDVI) since it is generally strongly correlated with climatic parameters (precipitation and temperature) and soil moisture content. These factors directly or indirectly influence host-parasite relationships and the propagation of environmentally transmitted helminths and may be important driver of between population variation in infection patterns.

The African elephant utilizes a wide range of habitats and lives in socially structured contact networks. Within elephant populations, individual animals live in structured groups that exhibit fission-fusion dynamics varying between sex-age groups [15–18]. Females and their offspring form fusion-fission matriarchal social groups where adult females and their calves live in stable units that coalesce with other similar cow-calf groups to create family and bond groups, thereby allowing adult females to form a nested or hierarchical social structure [15]. Males, on the other hand, form fluid social groups of mixed or similarly aged males in bachelor groups that have periodical contact with matriarchal groups when searching for mating opportunities [16]. Due to these more fluid social dynamics and greater mobility, males rove more widely than females [19] and adult males have larger home ranges than the immature males that still form part of the family groups [20–22].

The aims of this study were to examine helminth infection patterns between and within the most important elephant populations in Kenya found in a number of different agro-ecological zones, and to test the importance of influence of age, social structure and NDVI as drivers of these infection patterns.

## Methods

### Study area

**Fig. 1:**
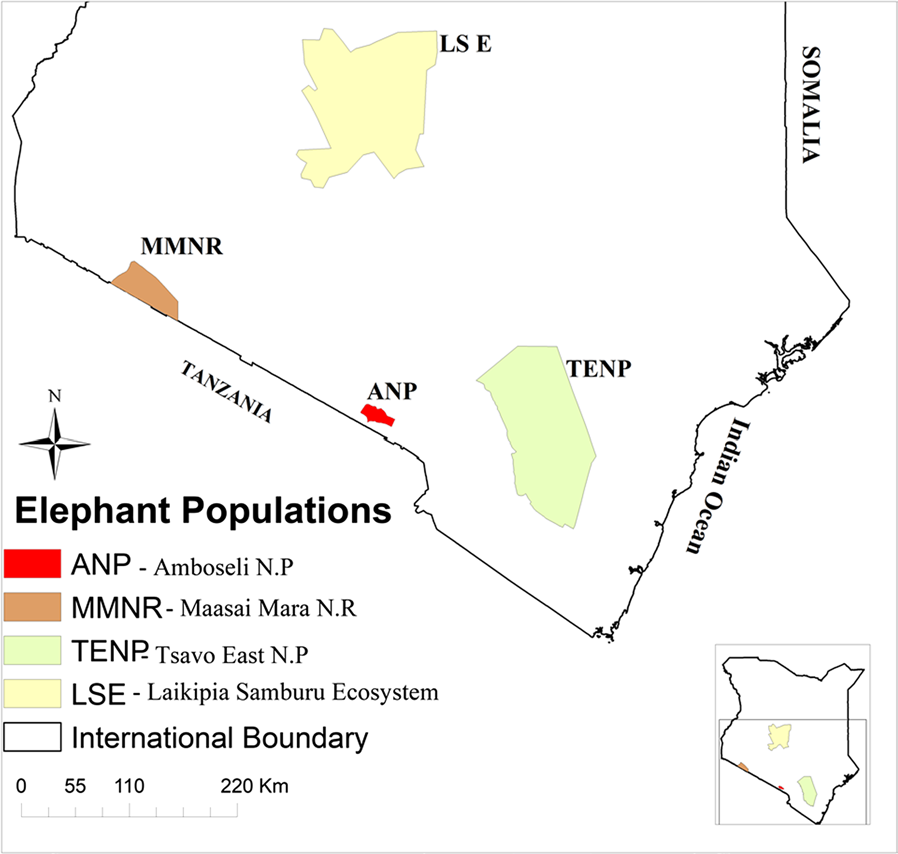
Map of Kenya showing the locations of the four major elephant populations in Kenya

The study was carried out in Tsavo East National Park, Laikipia-Samburu Ecosystem, Maasai Mara National Reserve and Amboseli National Park (Fig. 1), the four conservation areas that hold the largest elephant populations in Kenya. These populations remain separate and do not mix.

Tsavo East National Park (TENP) is situated in south-east of Kenya and enjoys a semi-arid savannah climate with a bimodal annual rainfall pattern. Heavy rains occur in April-May, while light rains fall in November-December. Overall, rainfall is erratic and low, with an annual average of 300–600 mm. This area holds 7727 elephants according to the 2018 large mammal census conducted by the Kenya Wildlife Service. Laikipia-Samburu Ecosystem (LSE) is in central Kenya and is covered by arid savannah grassland with annual rainfall of 300–700 mm. Rainfall is bimodal and falls in April-May and November-December. The Laikipia-Samburu Ecosystem hosts an elephant population estimated at 7166 during the 2017 large mammal census carried out by Kenya Wildlife Service. The Maasai Mara National Reserve (MMNR) is located in southern Kenya close to the border between Kenya and Tanzania, where it is contiguous with the Serengeti. Overall, this area consists of a large expanse of savannah grassland with annual rainfall ranging from 650 mm in the south-east to about 1300 mm in the north-west. Most rain falls in March-May, although some also falls in October-November. The 2017 large mammal census reported 2493 elephants in this national reserve. Lastly, Amboseli National Park (ANP) is located at the base of Mount Kilimanjaro in southern Kenya. It holds 2127 elephants according to the 2018 large mammal census report by the Kenya Wildlife Service. It consists mostly of arid dry savannah open grassland land, mixed with patches of scrub and *Acacia xanthophloea* woodland. Average annual rainfall is 340 mm with an annual range of 141–757 mm (https://amboselibaboons.nd.edu/downloads/). A network of marshes fed by underground water originating as snow melt from Mount Kilimanjaro provides a permanent water supply.

### Faecal sampling

Faecal sampling was carried out in February-November 2017 using a cross-sectional study design. Individuals in a social family herd, male bachelor herds and lone bulls were tracked until they defecated. From each animal defecation, a fresh dung bolus was carefully opened and approximately 20 g of the dung were scooped out and preserved in 10% formalin. The following information was recorded for each sample: age of the individual animal (adult, subadult or juvenile), sex, date of collection, GPS coordinates at the time of sighting, and type of social group (whether part of a female or male social group). A female social group was defined as a group consisting of females and their offspring and occasional males, whereas a male social group was taken to be as a solitary male or a group of two or more males seen in proximity at the time of observation. A total of 243 faecal samples, 71 from independent male groups or solitary males and 172 from family social groups, were collected in the four study areas. Totals of 62 family groups were sampled in MMNR, 37 in TENP, 27 in ANP and 19 in LSE, while 19 male social groups were sampled in MMNR, 22 in TENP, 16 in ANP and 14 in LSE.

### Coprological analyses

#### Sedimentation technique

A method described by VanderWaal et al. [23] with a slightly modified procedure was applied. Approximately 4 g of dung was weighed, mixed with 45 ml of tap water in a 50 ml centrifuge tube, and stirred until the mixture became a slurry. The dung slurry was then sieved and left to stand for 30 min. Decanting and re-suspension of the sediment was repeated 2–3 times until the suspension cleared. A dropping pipette was used to place ~ 0.05 ml of the sediment on a glass slide for examination under a Leica DM500 microscope. The presence of nematode and trematode eggs was assessed and micrographs of at least 10 eggs of different morphotypes were taken. Measurements of eggs (length and width) were taken from the photomicrographs using Leica LAS EZ software (Leica Microsystems GmbH, Wetzlar, Germany).

#### Flotation technique

Faecal samples were thoroughly homogenized with a stirring stick so that parasite eggs would be uniformly distributed throughout the sample. Initially, a faecal floatation fluid with specific gravity of 1.27 was prepared. Briefly, 454 g of table sugar was weighed and mixed with 355 ml of distilled water. The mixture was heated over low heat whilst being stirred until all the sugar had dissolved. The sugar solution was left to cool before use as the floatation fluid. Faecal samples were homogenized (as in the sedimentation technique) and prepared by weighing approximately 4 g of the elephant dung. The sample was mixed with 12 ml tap water, stirred and sieved through a tea strainer, before being transferred to a 15 ml plastic centrifuge tube. If the filtrate was less than 15 ml, it was topped up with tap water and the tube capped. It was then centrifuged at 1500× *rpm* for 10 min. The supernatant was decanted out and the sediment re-suspended using the flotation fluid to fill up half the test tube. The sediment was mixed thoroughly with the flotation fluid using a stirring stick. The tube was then filled to the top with more flotation fluid until a slight bulging meniscus formed. A coverslip was gently placed on the centre of the top of the tube. The tubes were then centrifuged for 10 min at 1500× *rpm*. After centrifugation, the coverslip was gently removed and placed directly onto a clean glass slide for examination under the microscope. Helminth eggs were qualitatively assessed. Photomicrographs of at least 10 eggs of different morphotypes were taken and processed as described in the sedimentation section.

#### McMaster technique

The helminth eggs were counted using a quantitative technique based on a calibrated McMaster chamber. Egg counts give an estimate of the number of eggs per gram (epg) in the faecal sample. The faecal sample was prepared as described for the floatation technique. A pipette was used to transfer the mixture to each of the two chambers of the McMaster slide. The preparation on the slide was left to settle for at least 5 min and then examined under the microscope. The eggs present in each chamber were counted. The total count for the slide was multiplied by a constant (50) to give the number of eggs per gram.

### Normalized difference vegetation index (NDVI) analysis

NDVI is a measure of reflectance and absorbance of the light spectra by vegetation and depends on the phenology and density of the vegetation being tested. Green vegetation reflects mostly green and near-infrared light spectra and absorbs red and blue light spectra and is often used as an index of productivity as it is correlated to plant phenology and nitrogen content. Overall, NDVI is strongly influenced by climatic parameters such as precipitation and temperature, and by soil moisture. These factors directly or indirectly influence host-parasite relationships and the transmission of environmentally transmitted helminths.

NDVI data were taken from satellite images obtained from a Landsat 8 satellite using the Operational Land Imager and Thermal Infrared Sensors for data capture. Satellite images of 30 m resolution were retrieved from the Libra development seed website (https://developmentseed.org/projects/libra/). Shape files of the four study areas were obtained from the Kenya Wildlife Service and were used to resize the relevant satellite images. The Amboseli satellite images were downloaded from 168 path and 062 row in April-November 2017; images for Maasai Mara were downloaded from 169 path and 061 row in December 2016 and January-March 2017; images for Laikipia-Samburu were downloaded from 168 path and 060 row in April-August 2017; and images for Tsavo East were downloaded from 167 path 062 row and from 163 path and 062 row in October-December 2016 and January-March 2017. These periods coincided with the sampling months and the three months prior to sampling. The Tsavo East satellites images were mosaicked into a single image using Q GIS software (Creative Commons, Mountain View, USA).

Selected satellite images were pre-processed using Q GIS to remove both radiometric and geometrical errors. The corrected images were used to generate the normalized difference vegetation index (NDVI) at a resolution of 250 × 250 m from 100 randomly selected points in each protected area. The final NDVI was calculated using the equation: NDVI = (NIR − RED)/(NIR + RED), where NIR represents the near-infrared electromagnetic ray and RED the visible red ray. Given that NDVI is a standardized method used to evaluate the health status of vegetation by quantifying the difference between the near-infrared electromagnetic ray (which vegetation strongly reflects) and the visible red light (which vegetation absorbs), the formula generates values between − 1 and + 1: negative values indicate the presence of water, while values close to 0 indicate bare soils; values between 0.1 and 0.5 indicate low-to-medium vegetation density cover, while values between 0.5 to + 1 indicate high vegetation density. To generate the NDVI raster, the calculator tool in Q GIS was used. As well, random points were generated within the study area, for which the NDVI values were extracted. This was done because there was an assumption that elephants move within their habitats. The NDVI values generated for the random points were compared to the actual sampled elephant locations for both the families and males recorded in the field.

### Statistical analyses

Assigning eggs of strongylid nematodes into taxonomic classes using measurements is a challenge as there is some degree of overlap in the dimensions of the eggs of these taxa. Moreover, when the measurements are multidimensional, discordance in measurements taken in a one dimension can lead to biases when assigning eggs to genera. However, by using model-based clustering, these problems can be overcome as information on the variation in the densities of the measurements across the taxa and the covariance of the different measurements is used to minimize the assignment bias. We employed an unsupervised multivariate cluster model using Gaussian finite mixture analysis to group nematode and trematode eggs into operational taxonomic units (OTUs) using egg measurements. The Gaussian finite mixture model (GMM), assumes that the measurements of helminth eggs taken from each taxon (species or genus) will follow a normal distribution resulting in a (multivariate) Gaussian distribution; each taxonomic component will form a cluster of unique density, centred at the mean vector, and with other geometric features such as volume, shape and orientation of the measurements determined by the covariance matrix. The volume, shape and orientation of the covariance’s can be constrained to be equal or variable across groups, giving rise to 14 possible models characterized by unique geometric characteristics [24]. The most parsimonious parameterisation of the covariance matrix is obtained using eigen-decomposition. The Gaussian finite mixture clustering process provides a model estimate for the data that allows for overlapping clusters and produces a probabilistic clustering that quantifies the uncertainty of observations belonging to the components of the mixture. The unsupervised GMM was performed using the *mclust* package [24] of the R statistical software [25]. The OTUs of the nematode and trematode eggs were assigned to taxonomic classes of helminth eggs based on mean length and width measurements taken from published records (Additional file 1: Table S1). We present here the data at the generic rather than the species level due to the variability in helminth egg sizes reported in previous studies.

To test our hypotheses, we conducted both bivariate and multivariate analyses to evaluate whether the variation in the dependent covariates such as helminth prevalence and helminth epg are influenced by independent covariates such as NDVI and sociality in the presence of unbalanced data. Any discordance in bivariate and multivariate models suggests that imbalance in data is causing spurious partial covariate effects. Using bivariate analyses and Friedman and Kruskal-Wallis tests, we tested for differences in epg between populations. We conducted multivariate analyses using Poisson and negative binomial generalized linear models (GLM) including hurdle and simple count models. The best model was selected based on parsimony criteria using Akaike information criteria (AIC). To examine the influence of social group type, NDVI and age on epg, we used the negative binomial hurdle GLM with the glmmTMB package [26] in the R statistical software [25].

## Results

**Fig. 2:**
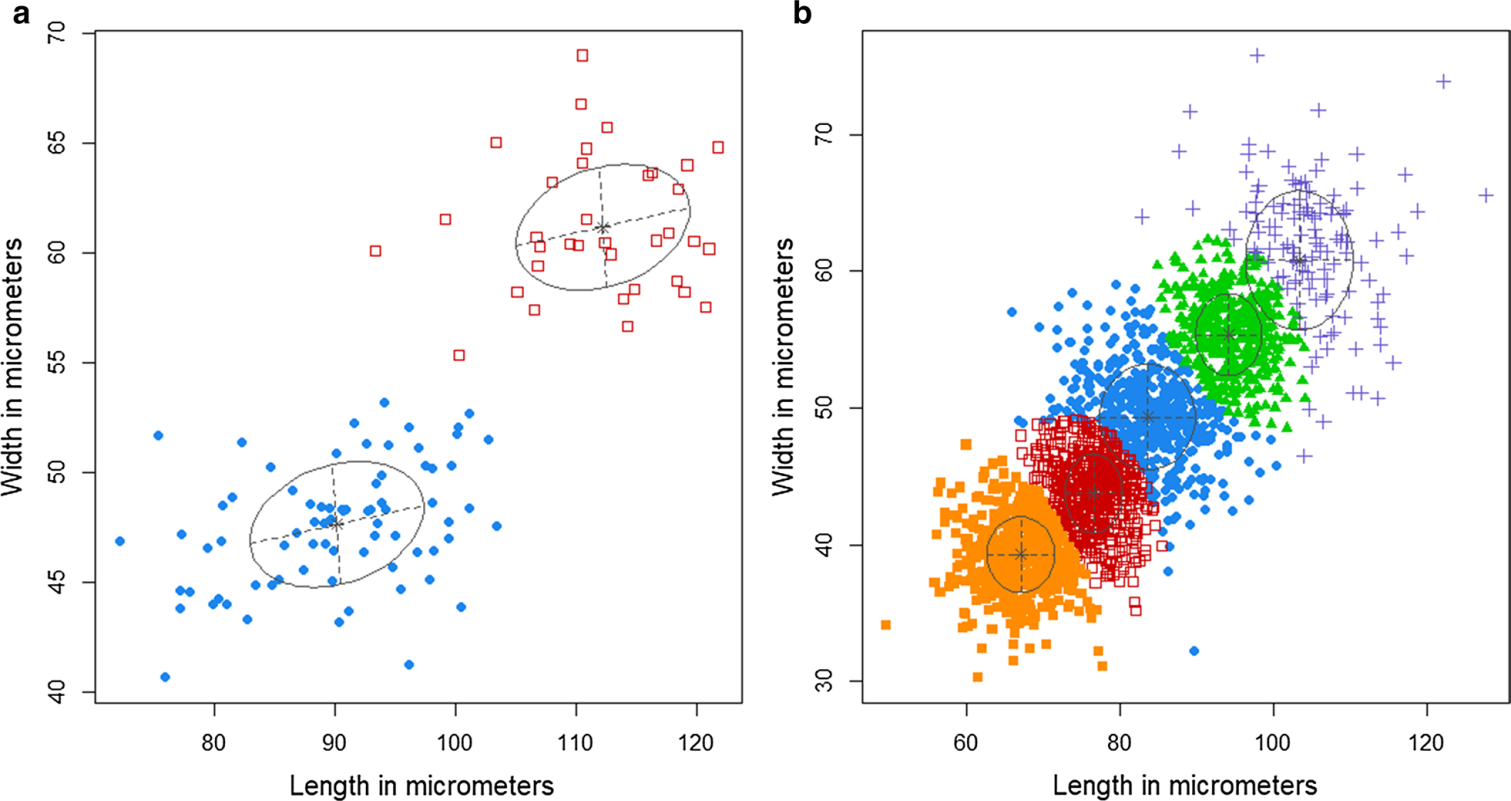
Model-based classification into operational taxonomic groups of elephants’ **a** trematode and **b** Strongylidae (nematode) eggs. Trematodes are classified into two (OTU1, blue; OTU2, red) and nematodes into five (OTU1, green; OTU2, orange; OTU3, purple; OTU4, red; and OTU5, blue) operational taxonomic units

**Fig. 3:**
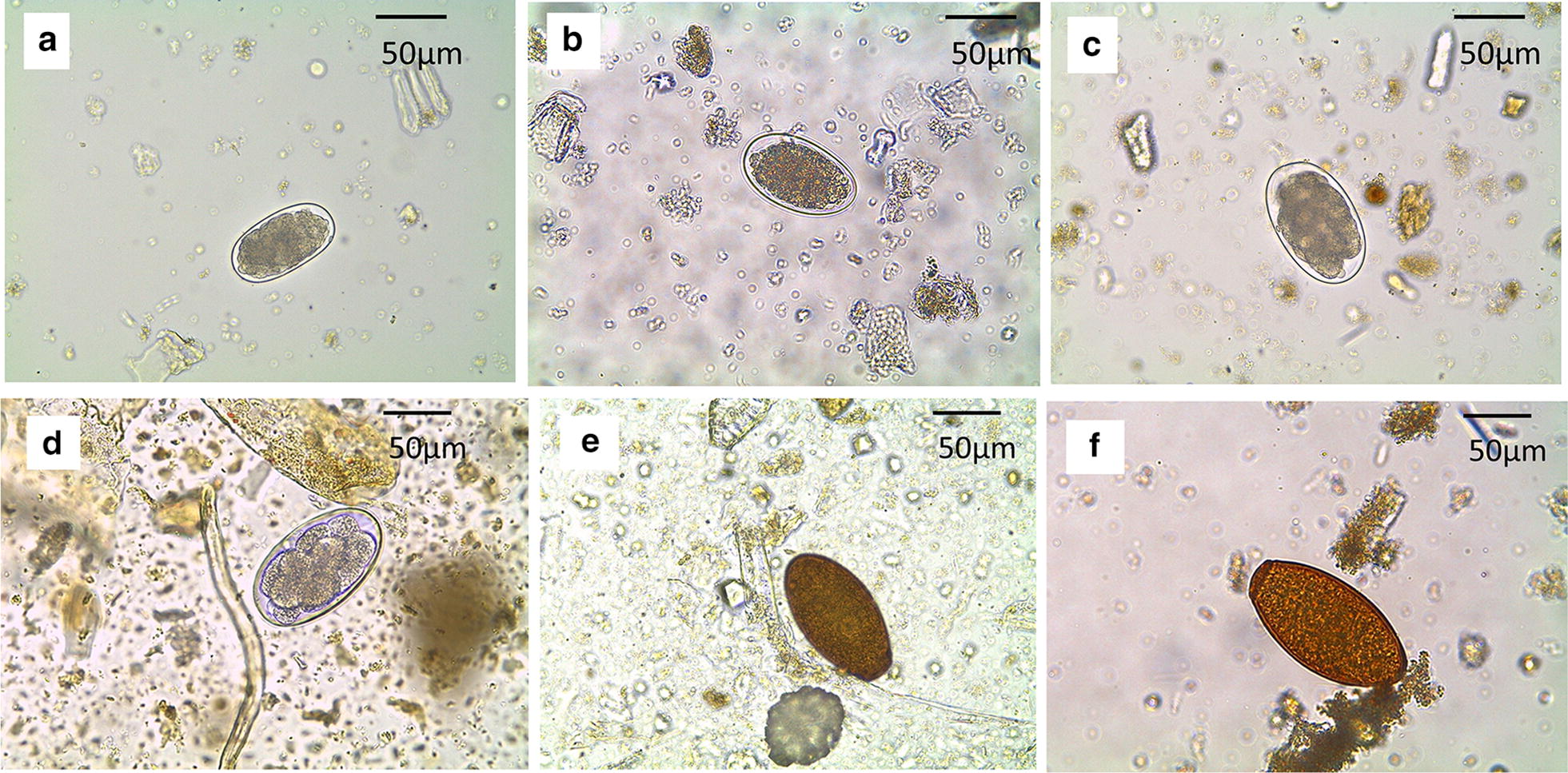
Photomicrographs of eggs of nematodes and trematodes of five genera. **a***Murshidia* (69 *×* 37 µm). **b**, **c** Different egg sizes of *Quilonia*: 96 × 57 µm (**b**) and 84 × 52 µm (**c**). **d***Mammomonogamus* (101 × 59 µm). **e***Protofasciola* (90 × 49 µm). **f***Brumptia* (115 × 59 µm)*. Scale-bars*: 50 µm

The best Gaussian finite mixture cluster model for trematodes was one with two components characterized by ellipsoidal, equal volume, shape and orientation (EE2) (Fig. 2). This model revealed that elephant populations in Kenya were infected by trematodes that can be characterized by two OTUs. Based on published egg dimensions of trematodes infecting African elephants, we found that trematode OTU1 had mean egg lengths and widths that were similar to those for *Protofasciola robusta*, while OTU2 had egg dimensions that were similar to *Brumptia bicaudata* (Figs. 2, 3; Table 1, Additional file 1: Table S1). For nematodes, the best supported model based on BIC consisted of five diagonal components with equal shape [27] indicating the presence of five OTUs (Fig. 2, Table 1, Additional file 1: Table S1): one group putatively belongs to the genus *Murshidia* (OTU1) with similar egg measurements to *Murshidia dawoodi*; three groups belong to the genus *Quilonia* (OTU2, OTU3 and OTU4) (Fig. 3), with egg measurements very similar to those of *Quilonia apiensis* (OTU2), *Q. africana* (OTU3) and *Q. magna* (OTU4). Finally, OTU5 had large egg measurements went beyond the range for the genus *Quilonia* but were similar to those recorded for *Mammomonogamus loxodontis* (Table 1, Additional file 1: Table S1).

**Table 1:** Results of unsupervised classification of trematode and nematode eggs into operational taxonomic units (OTUs)

OTUs	Mean ± SD		Percentile (2.5–97.5%)		Range		*n*
	Length (µm)	Width (µm)	Length (µm)	Width (µm)	Length (µm)	Width (µm)	
Trematode OTUs							
OTU1	90 ± 7	48 ± 3	76–101	43–52	72–103	41–53	76
OTU2	113 ± 7	61 ± 3	98–122	56–68	93–122	55–69	36
Nematode OTUs							
OTU1	67 ± 4	39 ± 3	58–74	34–44	50–78	30–47	492
OTU2	83 ± 5	49 ± 3	72–94	43–57	66–100	32–59	452
OTU3	76 ± 3	43 ± 2	70–82	38–48	67–85	35–49	556
OTU4	94 ± 4	55 ± 3	87–102	50–61	85–105	48–62	441
OTU5	105 ± 6	62 ± 5	90–117	51–71	83–128	46–76	125

*Abbreviation*: SD, standard deviation

The prevalence of infection determined from sedimentation was 97.5%, whereas the prevalence obtained from floatation was 92.6%; this difference, however, was not statistically significant (*χ*_(1, *n* = 243)_^2^ = 0.769, *P* = 0.366; Table 2). Therefore, all analyses of the prevalence were based on results obtained using the sedimentation technique. The prevalence of helminth infection determined using sedimentation varied between populations and was statistically significant (*χ*_(3, *n* = 243)_^2^ = 8.972, *P* = 0.030); however, there was no association between prevalence and elephant social group (male social groups *vs* female social groups, *χ*_(1, *n* = 243)_^2^ = 0.461, *P* = 0.497). The prevalence of nematodes was 96.3% (95% CI: 93.09–98.29%) and was significantly higher than that of trematodes, which was 39.1% (95% CI: 32.92–45.54%; *χ*_(1, *n* = 243)_^2^ = 179.18, *P* < 0.001). There was no significant influence of either social group (*χ*_(1, *n* = 243)_^2^ = 1.952, *P* = 0.162) or sampling location (*χ*_(3, *n* = 243)_^2^ = 5.956, *P* = 0.114) on nematode prevalence (Table 3). By contrast, trematode prevalence was significantly influenced by the location and elephant population (*χ*_(3, *n* = 243)_^2^ = 53.13, *P* < 0.001, Table 2) but not by social group (*χ*_(1, *n* = 243)_^2^ = 0.254, *P* = 0.614; Table 3).

**Table 2:** Variation in the prevalence of helminths in elephant populations and social groups in Kenya estimated using sedimentation and floatation methods

Elephant population	*n*	Floatation	Sedimentation
Male social group			
Amboseli	16	88	94
Laikipia-Samburu	14	86	100
Maasai Mara	19	100	100
Tsavo East	22	86	91
Total	71	90	96
Family social group			
Amboseli	27	93	96
Laikipia-Samburu	46	98	100
Maasai Mara	62	100	100
Tsavo East	37	78	95
Total	172	94	98
Male and family social groups combined			
Amboseli	43	91	95
Laikipia-Samburu	60	95	100
Maasai Mara	81	100	100
Tsavo East	59	81	93
Total	243	93	98

**Table 3:** Prevalence of nematodes and trematodes in male and family social groups in different populations estimated using the faecal sedimentation method

Elephant population	*n*	Trematodes (%)	Nematodes (%)
Male social group			
Amboseli	16	44	94
Laikipia-Samburu	14	79	93
Maasai Mara	19	42	100
Tsavo East	22	18	86
Total	71	42	93
Family social group			
Amboseli	27	41	96
Laikipia-Samburu	46	76	100
Maasai Mara	62	19	98
Tsavo East	37	19	95
Total	172	38	98
Male and family social group combined			
Total	243	39	96

**Fig. 4:**
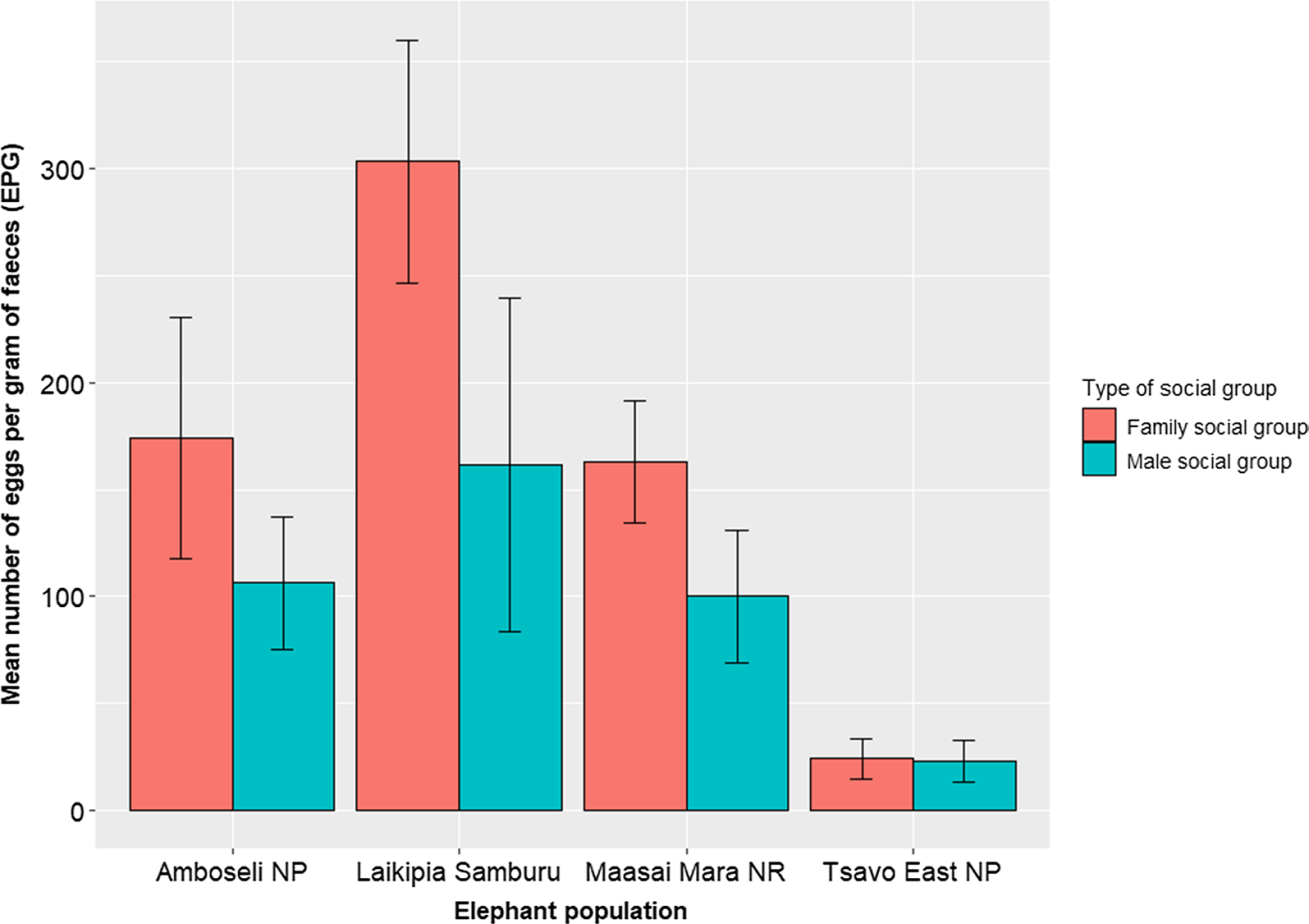
Mean egg burden (epg faeces) of helminths for each social group in the studied Kenyan elephant populations

The quantitative analysis using the McMaster technique revealed that the mean epg varied within elephant populations and between elephant social groups. Bivariate analyses revealed that elephants sampled in family groups had significantly higher median epg than solitary males and/or males in bachelor groups when controlling for epg variation across sampling locations or elephant populations (Friedman *χ*_(1, *n* = 243)_^2^ = 4, *P* = 0.046; Fig. 4). Elephant family groups in the four elephant populations showed significant differences in mean epg (Kruskal-Wallis *χ*_(3, *n* = 243)_^2^ = 40.942, *P* < 0.001; Fig. 4). Similarly, male groups from various elephant populations differed in their mean epg (Kruskal-Wallis *χ*_(3, *n* = 243)_^2^ = 9.38, *P* = 0.025; Fig. 4). Young males in mixed sex family groups had a lower epg than that of females (Table 4) when controlling for population or age; however, the differences were not statistically significant (population: Friedman *χ*_(1, *n* = 243)_^2^ = 1, *P* = 0.317; age: Friedman *χ*_(1, *n* = 243)_^2^ = 2, *P* = 0.157).

**Table 4:** Mean helminth burden (epg faeces) for each sex and type of social group in Kenyan elephant populations

Sex and social group	*n*	Mean ± SD	Median
Amboseli elephants			
Females in a family social group	19	202.63 ± 318.62	50
Males in a family social group	7	121.43 ± 236.04	50
Males in a male social group	16	106.25 ± 125.00	75
Laikipia-Samburu elephants			
Females in a family social group	35	320.00 ± 418.89	200
Males in a family social group	4	275.00 ± 332.92	175
Males in a male social group	14	171.43 ± 272.25	50
Maasai Mara elephants			
Females in a family social group	46	145.65 ± 204.62	100
Males in a family social group	8	200.00 ± 276.46	100
Males in a male social group	19	89.47 ± 132.89	50
Tsavo East elephants			
Females in a family social group	25	36.00 ± 66.96	0
Males in a family social group	10	0	0
Males in a male social group	22	22.73 ± 45.58	0

The normalized difference vegetation index (NDVI) was generally very low in the areas occupied by the study populations. The average NDVI over a three-month period varied between the four study locations and these differences were statistically significant (Kruskal-Wallis; *χ*_(3, *n* = 243)_^2^ = 9.18, *P* = 0.027). The lowest three-month mean NDVIs were recorded in Amboseli (mean ± standard deviation, 0.091 ± 0.002) and Tsavo East (0.118 ± 0.006) but were relatively higher in Laikipia-Samburu (0.16 ± 0.013) and Maasai Mara (0.239).

**Fig. 5:**
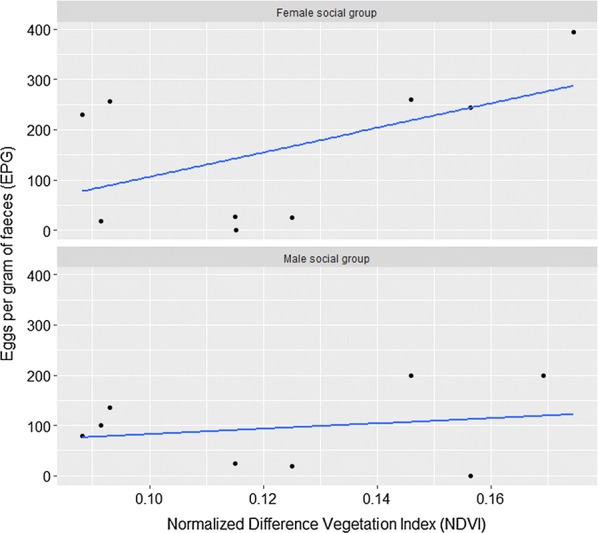
Scatterplot showing the relationship between NDVI and egg burden (epg faeces) for each social group for all elephant populations combined

**Fig. 6:**
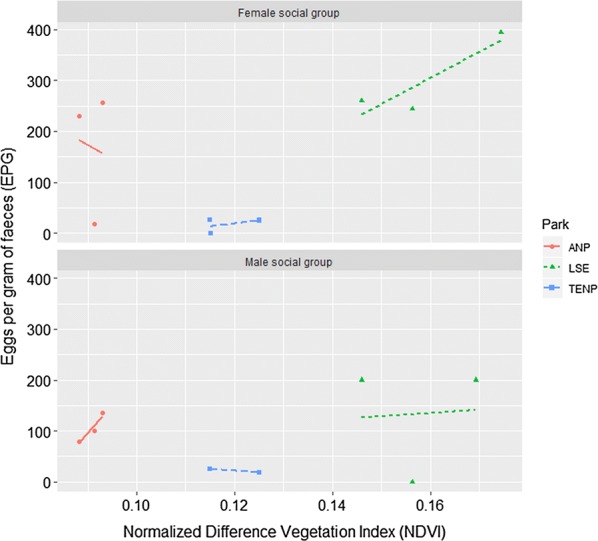
Scatterplot showing the relationship between NDVI and egg burden (epg faeces) for each social group for elephant populations treated separately

The most parsimonious multivariate model for variation in helminth epg was a hurdle GLM with a negative binomial distribution. This model indicated that the variation in non-zero positive counts of epg were driven by three-month cumulative mean NDVIs, social group type and elephant population, and sampling location (Table 5). We observed a positive association between mean epg and the three-month cumulative mean NDVI (Fig. 5). Among elephant social groups, female social groups had a higher mean epg than male social groups (Fig. 5). Among elephant populations or protected areas, the elephant populations in Samburu-Laikipia had a significantly higher epg than in Amboseli, while in Tsavo elephants had a significantly lower epg than in Amboseli (Fig. 6). However, Maasai Mara elephants showed no differences in their epg from Amboseli. In the binomial part of the model, which shows the presence or absence of a detectable epg, location and age and, to a lesser extent, NDVI all had a significant influence on detectable helminth infection. Laikipia-Samburu elephants had higher helminth prevalence than Amboseli elephants, whereas the elephants from Maasai Mara and Tsavo East had prevalence that were similar to Amboseli (Table 5). Adult elephants had a higher detectable epg than sub-adults and juveniles combined.

**Table 5:** A multivariate hurdle GLM showing important factors explaining variations in epg between Kenyan elephant populations

Covariate	Estimate	SE	*Z*-value	*P*-value
Count model coefficients (truncated negbin with log link)				
Intercept	3.09	0.92	3.35	0.001
3-month mean NDVI	24.18	10.04	2.41	0.016
Sub-adults and Juveniles *vs* Adults	0.11	0.18	0.63	0.528
Family social group *vs* male social group	0.29	0.18	1.68	0.094
Laikipia-Samburu *vs* Amboseli	− 1.43	0.72	− 2.00	0.046
Maasai Mara *vs* Amboseli	− 3.74	1.50	− 2.49	0.013
Tsavo East *vs* Amboseli	− 1.37	0.40	− 3.43	0.001
Log (theta)	0.38	0.11	3.41	0.001
Zero hurdle model coefficients (binomial with logit link)				
Intercept	5.27	2.69	1.96	0.050
3-month mean NDVI	− 56.56	29.46	− 1.92	0.055
Sub-adults and Juveniles *vs* Adults	− 0.71	0.36	− 1.96	0.050
Family social group *vs* male social group	0.72	0.36	2.01	0.045
Laikipia-Samburu *vs* Amboseli	5.04	2.21	2.28	0.023
Maasai Mara *vs* Amboseli	8.56	4.38	1.96	0.051
Tsavo East *vs* Amboseli	− 0.25	0.92	− 0.28	0.783

*Abbreviation*: SE, standard error

## Discussion

The African elephant is a mega herbivore and a keystone species in conservation whose ecological impact on the diversity and survival of habitats and other species is enormous [28]. Its numbers have continued to dwindle in many parts of its African range where populations are split into separate subpopulations [29]. As such, subpopulations may suffer different rates of parasite infestation. Here we present the first study to have examined helminth infection patterns in distinct African elephant populations, and the first to have evaluated factors associated with intra- and inter-population variability in prevalence and egg burden (epg). This study used only egg dimensions for helminth identification and the inference of prevalence and load since the obtaining of worms is often invasive or opportunistic. We identified two genera of trematodes, *Protofasciola* and *Brumptia*, and three of nematodes, *Murshidia*, *Mammomonogamus* and *Quilonia* (tribe Quiloninea, subfamily Cyathostominae). Using unsupervised classification of strongylid nematodes, we recovered OTUs that had egg dimensions corresponding to species that are known to occur in East Africa, in particular Kenya. Specifically, we recovered eggs from species including *P. robusta*, *B. bicaudata*, *Murshidia africana*, *Quilonia africana*, *Q. magna* and *Mammomonogamus loxodontis*, which demonstrates that, using information on egg measurements, it is possible to employ model-based clustering to group eggs into taxonomic units matching actual species. This type of model-based classification for nematode eggs has previously been tested with discriminations within the range of 80–95% for sheep nematodes [30, 31].

Although cluster-based modelling of the dimensions of strongylid-type eggs was used to identify genera and, potentially, species of the Strongylidae, the utility of OTUs detected by model-based clustering depends on the knowledge of egg measurements from species known to infect elephant populations. Such data will help identify any variation within species from different host populations, thereby providing useful information for egg identification through model clustering. A potential handicap with this method is that egg measurements for a single species taken from different host populations seem to vary greatly. The cause of this variation is not clear but could be due to the misidentification of larval nematodes from hatched eggs or to high inherent variance in nematode egg size that varies between host populations. The factors causing variance in egg measurements within species across host populations could hamper matching model based OTUs with known species. Thus, our species identification remains tentative and should not be taken as conclusive; nevertheless, it reveals the potential diversity in helminths that exists both within and between host populations.

The overall prevalence of helminths in elephants was 97.5%. However, this prevalence was characterized by a significantly higher proportion of nematodes (96.3%) than of trematodes (39.1%). In our study, we modified the sedimentation method commonly used for examining trematodes (which involves examining all of the sediment in a Petri dish under a dissecting microscope) [3]. However, our results were comparable with infection in elephants elsewhere, which suggests that our modifications did not significantly affect the sensitivity of the methodology. For instance, several studies have reported infection patterns in elephants in which nematode prevalence is 2–3 times greater than trematode prevalence. For instance, nematodes in elephant populations in Burkina Faso, West Africa, had an overall prevalence of 97.7% compared to 30.9% for trematodes [32]. A similar pattern has been observed in Botswana, South Africa, where elephants had a 100% prevalence of nematodes and 26% of trematodes [3]. This pattern is not restricted to the African elephant as a comparable prevalence of nematodes (92–96%) has been recorded in Asian elephants [33]. Furthermore, in our analysis, we observed significant inter-population variation in helminth prevalence. This was probably mainly due to trematode prevalence since, unlike nematodes, trematodes require the presence of an intermediate host (a snail) that depends on the presence of a permanent water source. Specifically, elephants from the Laikipia-Samburu ecosystem had the highest trematode prevalence, while elephants from Tsavo East had the lowest. By contrast, Amboseli elephants, that are known to be exposed to permanent water sources [34], had more moderate trematode prevalence than expected when compared to the Laikipia-Samburu population. The factors that determine trematode prevalence may be linked to the environmental variables influencing the abundance of snails, the intermediate hosts of trematodes [35]. As expected, the prevalence of trematodes in Amboseli elephants exposed to permanent water was nearly double that of elephants using the seasonally water-logged Okavango delta, where the prevalence of trematodes was 23% [3].

Mean egg burden was higher in family groups than in male social groups, which contradicts male-biased parasitism known to be associated with both hormonal and behavioural differences often seen in other animals. In cattle, younger animals and males tend to have higher levels of gastrointestinal parasite infection than older and female animals [36]. In most mammals, males exhibit higher infection rates than females (i.e. humans, ungulates, rodents, bats and birds) [27, 37–40]. The hormone testosterone is associated with immunosuppression in males, leading to greater parasite infection. However, Thurber et al. [1] found no effect of testosterone on parasite burden in male elephants in musth, individuals expected to have the highest level of testosterone. It is unlikely that testosterone will have more immunosuppressive effects on elephants than on other mammal species [3]. The higher parasite infection observed in female than in male elephants suggests that elephant social structures have a significant influence on mean egg burden since group-living exposes group members to higher parasite infection risks than individuals with solitary lifestyles or who form transient associations. This social dichotomy in infection patterns in elephants may be related to habitat use and ranging patterns, which drive the exposure and transmission of parasites such as helminths. In elephants, the ranging patterns of the female-led (matriarch) family groups are predictable as they often remain within reach of water, and long-distance movement is avoided due to the presence of juveniles. Solitary males or bachelor groups, on the other hand, have no such constraints and range over greater distances [19–22]. Moreover, infectious helminth propagules build up in frequently used habitats, hence family social groups suffer a higher risk of infection [41]. The influence of sociality on egg burden or egg shedding could also be attributed to the sex composition of the group, especially if the effects of male-biased parasitism are taken into account. However, we did not find any significant difference in egg burden between male and female individuals in the family social groups.

Although the influence of social structure on egg burden has been observed in other elephant populations, the egg burden detected by our study was much lower. We detected a mean egg burden of 172 in female groups and 89 in male groups, figures that are much lower than in elephant populations in the Okavango Delta, Botswana, where female groups had a mean egg burden of 1116 and males 529 [3]. A study of male elephants in Etosha National Park in Namibia revealed that in one year, the average strongylid egg burden varied between 1409–2204 in two different years [42]. In addition, previous studies on Rhodesian elephants have recorded much higher mean egg burden reaching 2072 with a range of 512–4382 [5]. Faecal egg counts or egg burden are often used to assess parasite burdens but have inherent pitfalls as they are subject to numerous variables that confound cause-effect relationships [43]. The few studies that have ever correlated egg burden to worm burdens have had variable outcomes [44–47]. Therefore, we believe that care should be taken with egg burden values we recorded from elephants since they may not correspond to the total worm burdens. A previous study has reported an average of around 30,000 worms per elephant with a range of 3837–105,294 [48]. The factors that influence variations in egg burden are not clear but may include factors intrinsic to these parasites including variations in the life histories of infecting worm species, the number of immature stages, worm sex imbalance and host-environmental factors [49].

Our results show that NDVI, a measure of vegetation productivity, biomass and habitat structure, were variable but generally low in all four studied habitats. However, NDVI was positively correlated with egg burden. Given that NDVI is correlated with environmental variables such as rainfall, soil moisture and habitat structure [50–54], it can both directly and indirectly determine the survival and transmission of infective stages, the maturation of immature worms in hosts, and the shedding rates of eggs by definitive hosts. Moreover, since NDVI is strongly correlated to primary production and the nutritional content of forage [55–59], it can also influence the distribution and abundance of the susceptible hosts and can enhance the heterogeneity of host spatial distribution [60–62]. Evidence of increased transmission of nematodes during the rainy season has been reported in a study of African elephants [3]. A study of human helminth infection also found a positive relationship between the prevalence of helminth infection and NDVI [63].

## Conclusions

Overall, our study shows that helminth infection in elephants is characterized by statistically significant inter-population variation in prevalence and egg burden. Sociality in elephants did not influence helminth prevalence but did have an influence on egg burden. Given that NDVI significantly varied between the four habitats and was positively correlated with mean egg burden it is likely that NDVI is an important driver of variation in egg burden in elephant populations.

## Supplementary information

**Additional file 1: Table S1.** Egg measurements (in µm) of gastrointestinal nematodes and trematodes infecting African elephants (data compiled from the literature).

## Data Availability

All data generated or analysed during this study are included in this published article and its additional file. Raw data used and/or analysed during the current study are available from the first and corresponding author upon request.

## Abbreviations

AIC: Akaikeʼs information criterion
ANP: Amboseli National Park
epg: eggs per gram
GLM: generalized linear model
GMM: Gaussian finite mixture model
LSE: Laikipia-Samburu ecosystem
MMNR: Maasai Mara National Reserve
OTU: operational taxonomic unit
TENP: Tsavo East National Park
NDVI: normalized difference vegetation index

## References

[1] Thurber MI, O’Connell-Rodwell CE, Turner WC, Nambandi K, Kinzley C, Rodwell TC (2011). Effects of rainfall, host demography, and musth on strongyle fecal egg counts in african elephants (*Loxodonta africana*) in Namibia. J Wildl Dis.

[2] Kinsella JM, Deem SL, Blake S, Freeman AS (2004). Endoparasites of African forest elephants (*Loxodonta africana cyclotis*) from the Republic of Congo and Central African Republic. Comp Parasitol.

[3] Baines L, Morgan ER, Ofthile M, Evans K (2015). Occurrence and seasonality of internal parasite infection in elephants, *Loxodonta africana*, in the Okavango delta, Botswana. Int J Parasitol Parasites Wildl.

[4] Condy JB (1974). Observations on internal parasites in Rhodesian elephant, *Loxodonta africana* Blumenbach, 1797. Proc Trans Rhodesia Sci Assoc.

[5] Van Der Westhuysen OP (1938). A monograph of the helminth parasites of the elephant. Onderstepoort J Vet Sc Anim Ind.

[6] Morgan ER, van Dijk J (2012). Climate and the epidemiology of gastrointestinal nematode infections of sheep in Europe. Vet Parasitol.

[7] Pandey V, Chitate F, Nyanzunda T (1993). Epidemiological observations on gastro-intestinal nematodes in communal land cattle from the highveld of Zimbabwe. Vet Parasitol.

[8] Pfukenyi DM, Mukaratirwa S (2013). A review of the epidemiology and control of gastrointestinal nematode infections in cattle in Zimbabwe. Onderstepoort J Vet Res.

[9] Froeschke G, Harf R, Sommer S, Matthee S (2010). Effects of precipitation on parasite burden along a natural climatic gradient in southern Africa—implications for possible shifts in infestation patterns due to global changes. Oikos.

[10] Apio A, Plath M, Wronski T (2006). Patterns of gastrointestinal parasitic infections in the bushbuck *Tragelaphus scriptus* from the Queen Elizabeth National Park, Uganda. J Helminthol.

[11] Turner WC, Getz WM (2010). Seasonal and demographic factors influencing gastrointestinal parasitism in ungulates of Etosha National Park. J Wildl Dis.

[12] Fowler ME, Mikota SK (2006). Biology, medicine, and surgery of elephants.

[13] Obanda V, Iwaki T, Mutinda NM, Gakuya F (2011). Gastrointestinal parasites and associated pathological lesions in starving free-ranging African elephants. S Afr J Wildl Res.

[14] Sherrard-Smith E, Chadwick EA, Cable J (2013). Climatic variables are associated with the prevalence of biliary trematodes in otters. Int J Parasitol.

[15] Wittemyer G, Douglas-Hamilton I, Getz WM (2005). The socioecology of elephants: analysis of the processes creating multitiered social structures. Anim Behav..

[16] Chiyo PI, Archie EA, Hollister-Smith JA, Lee PC, Poole JH, Moss CJ (2011). Association patterns of African elephants in all-male groups: the role of age and genetic relatedness. Anim Behav.

[17] Moss CJ, Poole JH, Hinde RA (1983). Relationships and social structure of african elephants. Primate social relationships: an integrated approach.

[18] Archie EA, Moss CJ, Alberts SC (2006). The ties that bind: genetic relatedness predicts the fission and fusion of social groups in wild African elephants. Proc R Soc B.

[19] Barnes RFW (1982). Mate searching behaviour of elephant bulls in a semi-arid environment. Anim Behav.

[20] Shannon G, Page B, Slotow R, Duffy K (2006). African elephant home range and habitat selection in Pongola Game Reserve, South Africa. Afr Zool.

[21] Leggett KEA (2006). Home range and seasonal movement of elephants in the Kunene region, northwestern Namibia. Afr Zool.

[22] Mills EC, Poulsen JR, Fay JM, Morkel P, Clark CJ, Meier A (2018). Forest elephant movement and habitat use in a tropical forest-grassland mosaic in Gabon. PLoS ONE.

[23] VanderWaal K, Omondi GP, Obanda V (2014). Mixed-host aggregations and helminth parasite sharing in an East African wildlife-livestock system. Vet Parasitol.

[24] Scrucca L, Fop M, Murphy TB, Raftery AE (2016). mclust 5: clustering, classification and density estimation using Gaussian finite mixture models. R J.

[25] R Core Team. R: A language and environment for statistical computing. Vienna: R Foundation for Statistical Computing; 2018.

[26] Magnusson A, Skaug H, Nielsen A, Berg C, Kristensen K, Maechler M, et al. glmmTMB: generalized linear mixed models using a template model builder. R package version 01. 2017;3.

[27] Guerra-Silveira F, Abad-Franch F (2013). Sex bias in infectious disease epidemiology: patterns and processes. PLoS ONE.

[28] Western D (1989). The ecological role of elephants in Africa. Pachyderm.

[29] Chase MJ, Schlossberg S, Griffin CR, Bouché PJ, Djene SW, Elkan PW (2016). Continent-wide survey reveals massive decline in African savannah elephants. PeerJ.

[30] Sommer C (1996). Digital image analysis and identification of eggs from bovine parasitic nematodes. J Helminthol.

[31] Christie M, Jackson F (1982). Specific identification of strongyle eggs in small samples of sheep faeces. Res Vet Sci.

[32] Nakandé A, Belem AMG, Nianogo AJ, Jost C (2007). Parasites gastro-intestinaux des éléphants dans la Réserve Partielle de Pama, Burkina Faso. Pachyderm.

[33] Vidya TNC, Sukumar R (2002). The effect of some ecological factors on the intestinal parasite loads of the Asian elephant (*Elephas maximus*) in southern India. J Biosci.

[34] Kioko J, Kiringe J, Omondi P (2006). Human-elephant conflict outlook in the Tsavo-Amboseli ecosystem, Kenya. Pachyderm.

[35] Lodge DM, Brown KM, Klosiewski SP, Stein R, Covich A, Leathers B (1987). Distribution of freshwater snails: spatial scale and the relative importance of physicochemical and biotic factors. Am Malacol Bull.

[36] Gunathilaka N, Niroshana D, Amarasinghe D, Udayanga L (2018). Prevalence of gastrointestinal parasitic infections and assessment of deworming program among cattle and buffaloes in Gampaha District, Sri Lanka. BioMed Res Int.

[37] Zuk M, McKean KA (1996). Sex differences in parasite infections: patterns and processes. Int J Parasitol.

[38] Martínez-Guijosa J, Martínez-Carrasco C, López-Olvera JR, Fernández-Aguilar X, Colom-Cadena A, Cabezón O (2015). Male-biased gastrointestinal parasitism in a nearly monomorphic mountain ungulate. Parasites Vectors.

[39] Connors V, Nickol B (1991). Effects of *Plagiorhynchus cylindraceus* (Acanthocephala) on the energy metabolism of adult starlings, *Sturnus vulgaris*. Parasitology.

[40] Krasnov BR, Bordes F, Khokhlova IS, Morand S (2012). Gender-biased parasitism in small mammals: patterns, mechanisms, consequences. Mammalia.

[41] Hausfater G, Meade BJ (1982). Alternation of sleeping groves by yellow baboons (*Papio cynocephalus*) as a strategy for parasite avoidance. Primates.

[42] Brumfitt KN. Assessment of body condition of African elephants (*Loxodonta africana*) in north east of Etosha National Park, Namibia: how it relates to strongyle parasite egg counts and nutrition value of feed. MSc. thesis, The University of Namibia, Windhoek, Namibia; 2015.

[43] Coulson G, Cripps JK, Garnick S, Bristow V, Beveridge I (2018). Parasite insight: assessing fitness costs, infection risks and foraging benefits relating to gastrointestinal nematodes in wild mammalian herbivores. Philos Trans R Soc B.

[44] Cripps J, Beveridge I, Martin JK, Borland D, Coulson G (2015). Temporal dynamics of helminth infections in eastern grey kangaroos (*Macropus giganteus*) in Victoria. Aust J Zool.

[45] Seivwright L, Redpath S, Mougeot F, Watt L, Hudson P (2004). Faecal egg counts provide a reliable measure of *Trichostrongylus tenuis* intensities in free-living red grouse *Lagopus lagopus scoticus*. J Helminthol.

[46] Gassó D, Feliu C, Ferrer D, Mentaberre G, Casas-Díaz E, Velarde R (2015). Uses and limitations of faecal egg count for assessing worm burden in wild boars. Vet Parasitol.

[47] Kim J-H, Choi M-H, Bae YM, Oh J-K, Lim MK, Hong S-T (2011). Correlation between discharged worms and fecal egg counts in human clonorchiasis. PLoS Negl Trop Dis.

[48] Condy JB (1974). Observations on internal parasites in Rhodesian elephants *Loxodonta africana* Blumenbach 1797. Proc Trans Rhod Sci Assoc.

[49] McKenna P (1981). The diagnostic value and interpretation of faecal egg counts in sheep. N Z Vet J.

[50] Gamon JA, Field CB, Goulden ML, Griffin KL, Hartley AE, Joel G (1995). Relationships between NDVI, canopy structure, and photosynthesis in three Californian vegetation types. Ecol Appl.

[51] Nicholson SE, Farrar TJ (1994). The influence of soil type on the relationships between NDVI, rainfall, and soil moisture in semiarid Botswana. I. NDVI response to rainfall. Remote Sens Environ.

[52] Farrar TJ, Nicholson SE, Lare AR (1994). The influence of soil type on the relationships between NDVI, rainfall, and soil moisture in semiarid Botswana. II. NDVI response to soil moisture. Remote Sens Environ.

[53] Davenport ML, Nicholson SE (1993). On the relation between rainfall and the normalized difference vegetation index for diverse vegetation types in East Africa. Int J Remote Sens.

[54] Li J, Lewis J, Rowland J, Tappan G, Tieszen LL (2004). Evaluation of land performance in Senegal using multi-temporal NDVI and rainfall series. J Arid Environ.

[55] Paruelo JM, Epstein HE, Lauenroth WK, Burke IC (1997). ANPP estimates from NDVI for the central grassland region of the United States. Ecology.

[56] Prince SD (1991). Satellite remote sensing of primary production: comparison of results for Sahelian grasslands 1981–1988. Int J Remote Sens.

[57] Tucker CJ, Vanpraet CL, Sharman MJ, Van Ittersum G (1985). Satellite remote sensing of total herbaceous biomass production in the Senegalese Sahel: 1980–1984. Remote Sens Environ.

[58] Albayrak S (2008). Use of reflectance measurements for the detection of N, P, K, ADF and NDF contents in sainfoin pasture. Sensors.

[59] Ryan SJ, Cross PC, Winnie J, Hay C, Bowers J, Getz WM (2012). The utility of normalized difference vegetation index for predicting African buffalo forage quality. J Wildl Manag.

[60] Sousa WP, Grosholz ED, Bell SS, McCoy ED, Mushinsky HR (1991). The influence of habitat structure on the transmission of parasites. Habitat structure; the physical arrangement of objects in space.

[61] Mueller T, Olson KA, Fuller TK, Schaller GB, Murray MG, Leimgruber P (2008). In search of forage: predicting dynamic habitats of Mongolian gazelles using satellite-based estimates of vegetation productivity. J Appl Ecol.

[62] Leimgruber P, McShea WJ, Brookes CJ, Bolor-Erdene L, Wemmer C, Larson C (2001). Spatial patterns in relative primary productivity and gazelle migration in the Eastern Steppes of Mongolia. Biol Conserv.

[63] Lai Y-S, Zhou X-N, Utzinger J, Vounatsou P (2013). Bayesian geostatistical modelling of soil-transmitted helminth survey data in the People’s Republic of China. Parasites Vectors.

