# Supplementary material for: Patterns of helminth infection in Kenyan elephant populations
Source: Parasit Vectors. 2020 Mar 18;13:145. doi: 10.1186/s13071-020-04017-1 (PMC7081694; doi:10.1186/s13071-020-04017-1)
Supplement: Supplementary file 1 — Additional file 1: Table S1. Egg measurements (in µm) of gastrointestinal nematodes and trematodes infecting African elephants (data compiled from the literature). [file 13071_2020_4017_MOESM1_ESM.docx]

**Additional file 1: Table S1.** Egg measurements (in µm) of gastrointestinal nematodes and trematodes infecting African elephants (data compiled from the literature).

| **Parasite** | **Length (µm)** | **Width (µm)** | **Length (µm)** | **Width (µm)** |
| --- | --- | --- | --- | --- |
| *Murshidia linstowi* | ^1^50 | 38 |  |  |
| *Murshidia longicaudata* | ^1^70 | 50 |  |  |
| *Murshidia hadia* | ^1^40 | 23 |  |  |
| *Murshidia africana* | ^1^62 | 32 | ^2^60 | 35 |
| *Murshidia omoensis* | ^1^60 | 30 |  |  |
| *Murshidia dawoodi* | ^1^65 | 35 |  |  |
| *Murshidia anisa* | ^1^48 | 30 |  |  |
| *Murshidia memphisia* | ^1^55 | 35 | ^3^73.25 | 40.82 |
| *Murshidia loxodontae* | ^1^60 | 35 |  |  |
| *Murshidia asiza* | ^1^60 | 35 |  |  |
| *Murshidia soudanensis* | ^1^60 | 35 |  |  |
| *Murshidia brevicaudata* | ^1^55 | 32 |  |  |
| *Khalilia* sameera | ^4^39 | 35 | ^8^57 - 78 | 38 - 40 |
| *Quilonia khalila* | ^1^75 | 38 |  |  |
| *Quilonia loxodontae* | ^1^82 | 44 | ^2^75 | 38 |
| *Quilonia magna* | ^1^80 | 45 |  |  |
| *Quilonia apiensis* | ^1^83 | 48 |  |  |
| *Quilonia africana* | ^1^73 | 30 | ^5^73 | 43 |
| *Quilonia uganda* | ^1^65 | 30 |  |  |
| *Quilonia brevicauda* | ^1^83 | 56 |  |  |
| *Quilonia ethiopica* | ^1^63 | 35 |  |  |
| *Mammomonogamus loxodontis* | ^6^100 | 40 |  |  |
| *Protofasciola robusta* | ^3^94.1 | 61.52 | ^7^87.7 | 48.5 |
| *Brumptia bicaudata* | ^2^114 | 76 |  |  |

Note: Sources of measurements are indicated as a superscript number and follow the order of the reference list provided below

References

1. Van Der Westhuysen OP. A monograph of the helminth parasites of the elephant. Onderstepoort Journal of Veterinary Science and Animal Industry 1938;10(1):49-190
2. Fowler ME, Mikota SK. Biology, Medicine, and Surgery of Elephants: Blackwell Publishing; 2006.
3. Condy JB. Observations on internal parasites in Rhodesian elephants *Loxodonta africana* Blumenbach 1797. Proceedings and Transactions of the Rhodesia Scientific Association. 1974; 55:67–99.
4. Monnig HO. On some Strongylid nematodes of the African elephant. Transactions of the Royal Society of South Africa. 1925 Jan 1;13(4):313-21.
5. Lane, C. Some Bursate Nematodes from the Indian and African Elephant. Ind. J1. Med. Res., 1921. vol. ix, No. 1, pp. 163-172.
6. Kinsella JM, Deem SL, Blake S, Freeman AS. Endoparasites of African forest elephants (Loxodonta africana cyclotis) from the Republic of Congo and Central African Republic. Comparative Parasitology. 2004;71 2:104-10.
7. Obanda V, Iwaki T, Mutinda NM, Gakuya F. Gastrointestinal parasites and associated pathological lesions in starving free-ranging African elephants. South African Journal of Wildlife Research. 2011;41(2):167-72. doi: 10.3957/056.041.0203.
8. Ogden CG. A revision of the genus Khalilia Neveu-Lemaire, 1924 (Nematoda: Strongyloidea). Parasitology. 1966 Aug;56(3):471-80.
